# Effects of Salinity on Abiotic Aggregation of Organic Matter and Subsequent Microbial Responses

**DOI:** 10.3390/gels8120836

**Published:** 2022-12-18

**Authors:** Tzong-Yueh Chen, Annelie Skoog

**Affiliations:** 1 Institute of Marine Environment and Ecology, National Taiwan Ocean University, Keelung 202, Taiwan; 2 Center of Excellence for the Oceans, National Taiwan Ocean University, Keelung 202, Taiwan; 3 Department of Marine Sciences, University of Connecticut, Groton, CT 06340, USA

**Keywords:** aggregation, particulate organic carbon, amino acids, neutral aldose, roller table

## Abstract

Studies of marine aggregation have focused on determining formation rates of larger particles from small particles. However, it has been shown that particles can form from the dissolved phase, which includes colloidal material. The purpose of this study was to investigate the effect of aggregation on the chemical composition of both the dissolved and particulate phases in two salinity regimes: (1) the coast of Avery Point, Connecticut, USA (AP; salinity of 30.1 psu); (2) the estuary of Thames River, Connecticut, USA (TR; salinity of 5.0 psu). The samples were incubated on a roller table for two days in the dark at a speed of 8 rpm. The mixed collision mechanism of shear and differential sedimentation provided by the roller table enhanced the gross aggregation of particulate organic carbon (POC; 0.75 µM d^−1^ and 1.04 µM d^−1^ in AP and TR, respectively). Subsequent microbial degradation led to a negative net aggregation of POC (−5.20 µM d^−1^ and −1.19 µM d^−1^ in AP and TR, respectively). Although bacterial abundance remained in a narrow range in this study, the aggregation of organic matter (OM) enhanced planktonic community respiration (CR; CR increased 5.1 mg-C m^−3^ d^−1^ and 205.4 mg-C m^−3^ d^−1^ in AP and TR, respectively). The collision also led to a gross aggregation of uncharacterized particulate organic matter (POM) transferred from uncharacterized dissolved organic matter (DOM; 0.62 µM-C d^−1^ and 0.56 µM-C d^−1^ in AP and TR, respectively). The aggregated, uncharacterized POM could be biologically refractory. The C- and N-yields and enrichment factor (EF) analysis indicated that the organic substrate dynamics in this study were complicated.

## 1. Introduction

Aggregation of organic matter (OM) in marine environments plays an important role in transformation processes of dissolved (and colloidal) organic substrates to the particulate form [1], as well as elemental cycling [2]. These processes could link the very large dissolved organic carbon (DOC) pool to the relatively small particulate organic carbon (POC) pool in the ocean. Marine colloidal gel is a subfraction of the empirically defined (by filtration) dissolved organic matter (DOM) pool. Colloidal material has a very large surface area and is known to play a critical role in aggregation [3,4]. The colloidal size fraction (also known as the high-molecular-weight size fraction) of naturally occurring OM contains a significantly higher concentration of chemically recognizable, biologically labile material than bulk DOM [4,5]. Specifically, colloidal OM has a high content of carbohydrates and amino acids [6,7], which may affect the aggregation processes [8]. Since colloidal material has a unique OM composition, it is possible that aggregation will change the chemical composition of both the dissolved and particulate pools by selectively transferring colloidal material from the dissolved to the particulate pool. 

In the open ocean, the intensive aggregation of OM is usually associated with a decline in phytoplankton blooms [9]. In coastal waters, the formation of organic aggregates could be more related to hydrodynamic processes, such as water turbulence and salinity change [10,11,12]. Mixing seawater in the estuary is generally considered to have a positive effect on the aggregation of OM due to the compression of the double layer of ions and the decrease in electrostatic repulsion between particles [13]. Salt-induced aggregation has been reported in various estuaries [14,15,16]. The maximum of the aggregation has been observed in the low to medium salinity (~15 psu), and the size of the aggregates decreased seaward [15,17].

Previous aggregation studies carried out in marine systems mainly focused on the characterization of the size, abundance, and chemical and microbial compositions of existing aggregates [18,19] or mathematical modeling based on particle size distributions [20,21]. Few studies have been carried out in which both aggregation and chemical OM characteristics have been determined [22,23,24,25,26]. Chen and Skoog [26] found that more POC was aggregated in brackish waters (salinity 6~7 psu) than in seawater samples (salinity 21~27 psu) in the eastern Long Island Sound, USA. However, only net aggregation was performed.

Here, our main goal of this study is to investigate changes in chemical characteristics in the dissolved and particulate phases in terms of both net and gross aggregation effects using a roller table in samples from one coastal site (Avery Point; denoted AP; salinity ~30 psu) and one estuarine site (Thames River, denoted TR; salinity ~5 psu) in the eastern Long Island Sound, USA. Bulk- and compound-specific concentration determinations (i.e., amino acids and neutral aldoses) were carried out in samples of both the dissolved and particulate phases.

## 2. Results and Discussion

### 2.1. Results

#### 2.1.1. In situ Conditions at AP and TR

The in situ salinities at AP and TR were 30.1 and 5.0, respectively, while the temperatures were 17.2 °C and 17.4 °C, respectively. The phosphate concentrations were 0.42 ± 0.05 µM and 0.25 ± 0.02 µM in AP and TR samples, respectively. In the AP samples, the dissolved inorganic nitrogen (DIN; sum of nitrate, nitrite, and ammonium) concentration was 3.01 µM (concentrations of nitrate, nitrite, and ammonium were 0.65 ± 0.01 µM, 0.16 ± 0.004 µM, and 2.20 ± 0.06 µM, respectively). In the TR samples, the DIN concentration was 19.80 µM (concentrations of ammonium, nitrate, and nitrite were 4.48 ± 0.78 µM, 15.19 ± 0.31 µM, and 0.13 ± 0.03 µM, respectively).

The inorganic N/P ratio in the initial samples from AP was 7.2, which is lower than the Redfield ratio of 16, indicating that the AP system was N-limited. In contrast, the inorganic N/P ratio in the initial samples from TR was 79.2, indicating that the system was highly P-limited.

#### 2.1.2. Changes in Bulk OM Concentrations in AP Samples

The initial POC and PON concentrations in AP were 28.97 ± 0.91 µM and 5.10 ± 0.22 µM, respectively, resulting in a particulate C/N ratio of 5.56 ± 0.11 (Table 1). POC and PON concentrations in the control were 17.07 ± 0.63 µM and 2.83 ± 0.28 µM, respectively, resulting in a particulate C/N ratio of 5.05 ± 0.43 (Table 1). POC and PON concentrations in the treatment were 18.57 ± 0.13 µM and 3.19 ± 0.18 µM, respectively, resulting in a particulate C/N ratio of 5.85 ± 0.30 (Table 1).

The POC concentrations in the control and treatment were both significantly lower than that in the initial samples (ANOVA, *p* < 0.05). However, the POC concentration in the treatment was significantly higher than that in the control (ANOVA, *p* < 0.05). The PON concentrations in the control and treatment decreased significantly from the initial (ANOVA, *p* < 0.05). The particulate C/N ratios were not different among the initial, control, and treatment samples (ANOVA, *p* > 0.05).

The initial DOC and TDN concentrations were 93.86 ± 4.96 and 7.07 ± 0.15 µM, respectively, resulting in a dissolved C/N ratio of 13.28 ± 0.42 (Table 1). DOC and TDN concentrations in the control were 89.94 ± 1.33 µM and 6.84 ± 0.76 µM, respectively, resulting in a dissolved C/N ratio of 13.28 ± 1.75 (Table 1). DOC and TDN concentrations in the treatment were 87.66 ± 4.27 µM and 6.15 ± 0.07 µM, respectively, resulting in a dissolved C/N ratio of 14.25 ± 0.77 (Table 1). There was no statistical difference in DOC concentrations, TDN concentrations, and dissolved C/N ratios among initial, control, and treatment samples (ANOVA, *p* > 0.05).

#### 2.1.3. Changes in Bulk OM Concentrations in TR Samples

The initial POC and PON concentrations were 24.24 ± 0.80 µM and 2.70 ± 0.14 µM, respectively, resulting in a particulate C/N ratio of 8.98 ± 0.41 (Table 1). POC and PON concentrations in the control were 19.86 ± 0.10 µM and 2.33 ± 0.10 µM, respectively, resulting in a particulate C/N ratio of 8.51 ± 0.20 (Table 1). POC and PON concentrations in the treatment were 21.87 ± 1.07 µM and 2.63 ± 0.11 µM, respectively, resulting in a particulate C/N ratio of 8.32 ± 0.24 (Table 1).

The POC and PON concentrations in the control were significantly lower than those in the initial (ANOVA, *p* < 0.05). Similar to AP, the bulk POC concentration in the treatment was significantly higher than that in the control, but it was lower than that in the initial (ANOVA, *p* < 0.05). The PON concentration in the treatment was significantly higher than that in the control (ANOVA, *p* < 0.05). The particulate C/N ratios had no significant difference among the initial, control, and treatment samples (ANOVA, *p* > 0.05). 

The DOC and TDN concentrations in the initial were 209.11 ± 5.38 µM and 18.59 ± 0.27 µM, respectively, resulting in a dissolved C/N ratio of 11.25 ± 0.35 (Table 1). The average bulk DOC and TDN concentrations in the control were 165.22 ± 15.91 µM and 14.54 ± 1.43 µM, respectively, resulting in a dissolved C/N ratio of 11.37 ± 0.24 (Table 1). Bulk DOC and TDN concentrations in the treatment were 128.88 ± 2.42 µM and 12.61 ± 0.28 µM, respectively, resulting in a dissolved C/N ratio of 10.36 ± 0.38 (Table 1).

Both DOC and TDN concentrations in the control were significantly lower than those in the initial (ANOVA, *p* < 0.05). The DOC concentration in the treatment was significantly lower than that in the initial and control (ANOVA, *p* < 0.05), whereas the TDN concentration in the treatment was only significantly lower than that in the initial (ANOVA, *p* < 0.05). The dissolved C/N ratio in the treatment was significantly lower than that in the initial and control (ANOVA, *p* < 0.05), indicating that bacteria consumed more DOC in the treatment compared to TDN.

#### 2.1.4. Changes in Bulk Amino Acids in AP Samples

The initial PHAA concentration was 811 ± 22 nM (Table 1), which is equivalent to 3.81 ± 0.12 µM-C and 1.00 ± 0.03 µM-N, accounting for 13.1 ± 0.4% and 19.7 ± 0.6% of POC (C-yields) and PON (N-yields), respectively. The PHAA concentration in the control was 725 ± 20 nM (Table 1), which is equivalent to 3.44 ± 0.13 µM-C and 0.91 ± 0.03 µM-N, accounting for 20.2 ± 0.7% and 32.0 ± 1.0% of POC and PON, respectively. The PHAA concentration in the treatment of AP was 785 ± 25 nM (Table 1), which is equivalent to 3.70 ± 0.13 µM-C and 0.97 ± 0.04 µM-N, accounting for 19.9 ± 0.7% and 31.6 ± 1.3% of POC and PON, respectively. 

The PHAA concentration in the control was significantly lower than that in the initial samples (ANOVA, *p* < 0.05). The PHAA concentration in the treatment was significantly higher than in control samples (ANOVA, *p* < 0.05). The PHAA C- and N-yields (representing PHAA contribution to POC and PON, respectively) were significantly higher in both the control and treatment than in the initial samples (ANOVA, *p* < 0.05).

The initial DHAA concentration was 766 ± 41 nM (Table 1), which is equivalent to 3.48 ± 0.30 µM-C and 0.94 ± 0.08 µM-N, accounting for 3.7 ± 0.2% and 13.2 ± 0.7% of DOC and TDN, respectively. The DHAA concentration in the control was 586 ± 35 nM (Table 1), which is equivalent to 2.68 ± 0.31 µM-C and 0.73 ± 0.08 µM-N, accounting for 3.0 ± 0.2% and 10.6 ± 0.4% of DOC and TDN, respectively. The DHAA concentration in the treatment of AP was 490 ± 27 nM (Table 1), which is equivalent to 2.23 ± 0.33 µM-C and 0.61 ± 0.09 µM-N, accounting for 2.5 ± 0.1% and 10.0 ± 0.7% of DOC and TDN.

The DHAA concentration, as well as the C- and N-yields, in the control was significantly lower than in the initial samples (ANOVA, *p* < 0.05), indicating the microbial degradation of DHAA. The DHAA concentration as well as the C-yield in the treatment was significantly lower than in both initial and control samples (ANOVA, *p* < 0.05), indicating either that aggregation enhanced the microbial DHAA consumption or selectively transferred DHAA to the particulate phase.

#### 2.1.5. Changes in Bulk Amino Acids in TR Samples

The initial PHAA concentration was 510 ± 18 nM (Table 1), which is equivalent to 2.36 ± 0.32 µM-C and 0.63 ± 0.02 µM-N, accounting for 9.7 ± 0.6% and 23.5 ± 1.1% of POC and PON, respectively. The PHAA concentration in the control was 431 ± 17 nM (Table 1), which is equivalent to 2.01 ± 0.08 µM-C and 0.54 ± 0.03 µM-N; 10.1 ± 0.4% and 23.3 ± 1.1% of POC and PON, respectively. The PHAA concentration in the treatment was 474 ± 28 nM (Table 1), which is equivalent to 2.20 ± 0.13 µM-C and 0.59 ± 0.03 µM-N, accounting for 10.0 ± 0.6% and 22.5 ± 1.6% of POC and PON, respectively.

The PHAA concentration in the control was significantly lower than that in the initial samples (ANOVA, *p* < 0.05), indicating the microbial degradation of PHAA. The PHAA concentration in the treatment was significantly higher than in control samples (ANOVA, *p* < 0.05), indicating the accumulation of PHAA in the treatment. PHAA There was no statistical difference in the C- and N-yields among the initial, control, and treatment samples (ANOVA, *p* > 0.05).

The initial DHAA concentration in TR was 626 ± 10 nM (Table 1), which is equivalent to 2.82 ± 0.23 µM-C and 0.78 ± 0.06 µM-N, accounting for 1.35 ± 0.02% and 4.2 ± 0.1% of DOC and TDN, respectively. The DHAA concentration in the control was 557 ± 9 nM (Table 1), which is equivalent to 2.51 ± 0.20 µM-C and 0.70 ± 0.05 µM-N, accounting for 1.52 ± 0.03% and 4.8 ± 0.1% of DOC and TDN, respectively. The DHAA concentration in the treatment was 507 ± 17 nM (Table 1), which is equivalent to 2.27 ± 0.20 µM-C and 0.63 ± 0.05 µM-N, accounting for 1.76 ± 0.07% and 5.0 ± 0.2% of DOC and TDN, respectively.

The DHAA concentration in the control was significantly lower than in the initial samples (ANOVA, *p* < 0.05), indicating the microbial degradation of DHAA. The DHAA concentration in the treatment was significantly lower than in the initial and control (ANOVA, *p* < 0.05), indicating either that aggregation enhanced the microbial consumption of DHAA or transfer of DHAA to the particulate phase. The C- and N-yields from DHAA in the control were higher than those in the initial (ANOVA, *p* < 0.05), indicating microorganisms selectively consumed other organic components rather than DHAA. The DHAA C-yield in the treatment was significantly higher than in the initial and control (ANOVA, *p* < 0.05), indicating that the aggregation reduced the degradation rate of DHAA.

#### 2.1.6. Changes in Bulk Neutral Aldose AP Samples

The initial PHNA concentration was 858 ± 37 nM (Table 1), which is equivalent to 5.00 ± 0.42 µM-C, comprising 17.2 ± 0.8% of POC. The PHNA concentration in the control was 615 ± 11 nM (Table 1), which is equivalent to 3.52 ± 0.25 µM-C, comprising 20.6 ± 0.3% of POC. The PHNA concentration in the treatment in AP was 571 ± 22 nM (Table 1), which is equivalent to 3.52 ± 0.25 µM-C, comprising 17.5 ± 0.7% of POC.

The PHNA concentration in the control was significantly lower than in the initial samples (ANOVA, *p* < 0.05), indicating the microbial degradation of PHNA. However, the C-yield from PHNA in the control of AP was significantly higher than that in the initial and treatment (ANOVA, *p* < 0.05). The PHNA concentration in the treatment was significantly lower than that in the initial and control (ANOVA, *p* < 0.05), indicating that the aggregation process enhanced the consumption of PHNA. 

The initial DHNA concentration was 2.37 ± 0.06 µM (Table 1), which is equivalent to 13.65 ± 0.48 µM-C, comprising 14.6 ± 0.4% of DOC. The DHNA concentration in the control of AP was 2.25 ± 0.04 µM (Table 1), which is equivalent to 12.96 ± 0.26 µM-C, comprising 14.4 ± 0.3% of DOC. The DHNA concentration in the treatment of AP was 2.15 ± 0.05 µM (Table 1), which is equivalent to 12.41 ± 0.28 µM-C, comprising 14.2 ± 0.2% of DOC.

The DHNA concentration in the control was significantly lower than that in the initial (ANOVA, *p* < 0.05), indicating the microbial degradation of DHNA. The DHNA concentration in the treatment was lower than that in the initial and control (ANOVA, *p* < 0.05), indicating that the aggregation process either enhanced the consumption of DHNA in the treatment or transferred from the dissolved to the particulate phase. The C-yield from DHNA in the control was lower than that in the initial (ANOVA, *p* < 0.05). The C-yield form of DHNA in the treatment was lower than that in the control and initial (ANOVA, *p* < 0.05). The decrease of C-yields in the control and treatment indicated that microorganisms selectively consumed DHNA rather than other DOC or that DHNA was selectively transferred from the dissolved to the particulate phase.

#### 2.1.7. Changes in Bulk Neutral Aldoses in TR Samples

The initial PHNA concentration was 617 ± 15 nM (Table 1), which is equivalent to 3.55 ± 0.22 µM-C and 14.6 ± 0.3% of POC. The PHNA concentration in the control was 444 ± 12 nM (Table 1), which is equivalent to 2.56 ± 0.22 µM-C and 12.9 ± 0.3% of POC. The PHNA concentration in the treatment was 572 ± 29 nM (Table 1), which is equivalent to 3.32 ± 0.32 µM-C and 15.2 ± 0.7% of POC.

The PHNA concentration in the control was significantly lower than in the initial samples (ANOVA, *p* < 0.05), indicating the microbial degradation of PHNA. The PHNA C-yield in the control was significantly lower than in the initial and treatment samples (ANOVA, *p* < 0.05), indicating selective microbial degradation of PHNA. The PHNA concentration in the treatment was significantly higher than in the control (ANOVA, *p* < 0.05), indicating the accumulation of PHNA in the treatment compared to the control. 

The initial DHNA concentration was 2.96 ± 0.09 µM (Table 1), which is equivalent to 17.26 ± 0.53 µM-C and 8.3 ± 0.3% of DOC. The DHNA concentration in the control was 2.80 ± 0.10 µM (Table 1), which is equivalent to 16.80 ± 0.58 µM-C and 10.2 ± 0.4%. The DHNA concentration in the treatment was 2.91 ± 0.06 µM (Table 1), which is equivalent to 16.95 ± 0.33 µM-C and 11.1 ± 0.2% of DOC. 

Contrary to AP, there was no statistical difference in DHAA concentrations among the initial, control, and treatment (ANOVA, *p* > 0.05). Although total concentrations of DHNA were similar, the C-yields significantly increased from the initial to the control and the treatment (ANOVA, *p* < 0.05), indicating selective degradation of DOC rather than DHNA.

#### 2.1.8. Compound-Specific Amino Acid Concentrations

In general, alanine (Ala), aspartic acid (Asp), glutamic acid (Glu), and glycine (Gly) were the most abundant amino acids in PHAA, both in AP and TR (Table 2). Asp was the most abundant amino acids in the particulate phase in AP (~13%), but Ala was the most abundant amino acids in TR (14–15%). These four amino acids comprised a narrow range of percentage in PHAA in three groups, both in AP and TR, which accounted for 46–48% of PHAA.

Similar to PHAA, Ala, Asp, Glu, and Gly were the most abundant amino acids in DHAA (Table 2). Asp was the most abundant DHAA in both AP and TR (13% and 15%, respectively). These four amino acids accounted for 45–47% and ~48% of DHAA in AP and TR, respectively.

#### 2.1.9. Compound-Specific Neutral Aldose Concentrations in AP Samples

In general, glucose and galactose were the most abundant PHNA. The particulate mol fractions of glucose and galactose in the initial were 35.8 ± 1.3% and 20.8 ± 0.7%, respectively (Table 3). The particulate mol fractions of glucose and galactose significantly decreased in the control and treatment (ANOVA, *p* < 0.05), indicating the selective consumption of glucose and galactose. The particulate mol fractions of neutral aldose other than glucose and galactose in AP increased in the control and treatment (ANOVA, *p* < 0.05) due to the huge mol fraction decrease of glucose and galactose.

Glucose was the most abundant DHNA in this study, which accounted for 24.9 ± 2.0% of DHNA in the initial of AP and 24.6 ± 0.8% and 24.9 ± 0.5% in the control and treatment, respectively (Table 3). Galactose was also abundant in DHNA in AP: the DHNA mol fraction of galactose ranged from 17.4 to 18.5%. There was no statistical difference in DHNA mol fractions among the initial, control, and treatment groups (ANOVA, *p* > 0.05).

#### 2.1.10. Compound-Specific Neutral Aldose Concentrations in TR Samples

The PHNA mol fractions of glucose and galactose in the initial samples were 31.8 ± 1.4% and 15.0 ± 0.8%, respectively (Table 3). Contrary to AP, the PHNA mol fractions of glucose and galactose significantly increased in the control and treatment (ANOVA, *p* < 0.05), indicating that glucose and galactose were biorecalcitrant. The PHNA mol fractions of aldoses other than glucose and galactose in the treatment were significantly lower than those in the initial samples (ANOVA, *p* < 0.05), indicating selective microbial degradation.

Glucose accounted for 37.4 ± 0.7% of DHNA in the initial samples and 38.4 ± 1.6% and 35.6 ± 0.8% in the control and treatment, respectively (Table 3). Glucose, mannose, and galactose accounted for 64.2 to 65.4% of DHNA. The DHNA mol fraction of glucose in the treatment was slightly but significantly lower than that in the initial and control (ANOVA, *p* < 0.05), indicating selectively microbial degradation of glucose or selective transfer of glucose to the particulate phase.

#### 2.1.11. Bacterial Abundance

Bacterial abundance in AP samples was 3.44 ± 0.95 × 10^5^ cell ml^−1^, 2.58 ± 0.59 × 10^5^ cell ml^−1^, and 2.25 ± 0.68 × 10^5^ cell ml^−1^ in the initial, control, and treatment, respectively. Bacterial abundance was not significantly different among the initial, control, and treatment samples from AP (ANOVA, *p* > 0.05). Using the bacterial cellular carbon content of 20 fg [27], this abundance equates to 0.57 ± 0.16 µM-C, 0.43 ± 0.10 µM-C, and 0.37 ± 0.11 µM-C of bacterial biomass in the initial, control, and treatment samples, respectively.

Bacterial abundance in TR samples was 7.21 ± 0.70 × 10^5^ cell ml^−1^, 5.81 ± 1.16 × 10^5^ cell ml^−1^, and 5.45 ± 0.99 × 10^5^ cell ml^−1^ in the initial, control, and treatment, respectively. Using the same bacterial carbon conversion factor of 20 fg bacterium^−1^ above [27], this abundance equates to 1.20 ± 0.12 µM-C. 0.97 ± 0.19 µM-C, and 0.91 ± 0.16 µM-C of bacterial biomass in the initial, control, and treatment samples, respectively. Similar to AP, bacterial abundance was not significantly different among the initial, control, and treatment from TR (ANOVA, *p* > 0.05).

### 2.2. Discussion

#### 2.2.1. Aggregation of Bulk Organic Matter

In this study, we evaluated the abiotic aggregation of OM through a roller table using the following two terms:

Net aggregation ≡ Treatment concentration − Initial concentration(1)

Gross aggregation ≡ Treatment concentration − Control concentration(2)

The net aggregation was negative in both AP and TR (−10.40 µM-C in AP and −2.37 µM-C in TR; Table 1). A negative net aggregation indicated that the amount of POC consumed by bacteria and dissociated by physical processes was larger than the amount of aggregated POC and associated bacterial biomass. Gross aggregation was positive in both AP and TR (+1.50 µM-C in AP and +2.08 µM-C in TR). A positive gross aggregation indicated that the collision mechanism provided by the roller table enhanced the aggregation of POC. Although we may observe a huge decrease in POC (−35.9% in AP and −9.8% in TR), the collision did enhance a significant gross aggregation of POC (+5.2% in AP and +8.6% in TR).

We are also interested in the fate of organic matter. A descriptive cartoon shows where the organic matter goes (see Figure 7 in [25]). In general, most disappearing organic matter in a closed system is due to respiration (paths 1 and 4). Aggregation processes may enhance the consumption of organic matter (paths 2 and 5). At the same time, aggregation processes can also transfer DOM to the particulate, which would lead to a concentration decrease in the dissolved phase and a concentration increase in the particulate phase (paths 3 and 6). In this cartoon, the disaggregation process was neglected because it occurs only in high turbulent conditions [28,29,30]. In AP experiment, 65.8% of disappeared DOC was transferred to POC and 34.2% of disappeared DOC was respired. In TR experiment, only 5.7% of disappeared DOC was transferred to POC, and most disappeared DOC was respired. The high percentage of respired DOC in TR indicated a more biologically-labile DOC in estuarine TR water. 

Bacteria and their subsequent colonization on aggregated materials may contribute to different fractions in POC. In this study, bacterial biomass contributed 2.0 to 2.5% of POC in AP, whereas it accounted for 4.2 to 5.0% of POC in TR. Note that the widely used bacterial carbon conversion factor in this study could be as much as a 40% underestimation of bacterial biomass in coastal environments [31].

#### 2.2.2. Changes in Biologically Labile Organic Components

Concentrations of PHAA in the control decreased from the initial, indicating the microbial degradation of PHAA. A positive gross aggregation of PHAA has been observed both in AP and TR, indicating that the accumulation of PHAA due to collision is stronger than the subsequent microbial consumption of PHAA. The gross aggregation of PHAA was 60 nM in AP and 43 nM in TR (Table 1), which accounted for 7.4% and 8.4% of the initial PHAA in AP and TR, respectively. The C- and N-yields of PHAA in the control and treatment of AP increased from the initial, indicating microorganisms preferentially used other POM rather than PHAA.

Concentrations of DHAA in the control decreased from the initial (Table 1), indicating the microbial degradation of DHAA. The concentration of DHAA in the treatment was lower than that in the control (Table 1), indicating either the transfer of DHAA to PHAA or an enhancement of the microbial degradation of DHAA during aggregation. The concentration difference of DHAA between the control and treatment in AP was 96 nM, and the aggregated PHAA in AP was 60 nM, indicating that the enhancement of microbial consumption of DHAA occurred with the aggregation of OM. The concentration difference of DHAA between the control and treatment in TR was 50 nM, and the aggregated PHAA in TR was 43 nM, indicating that the concentration difference of DHAA could be majorly transferred to the particulate phase. In AP experiment, 62.5% of disappeared DHAA was transferred to POC and 37.5% of disappeared DOC was respired. In TR experiment, 86.0% of disappeared DHAA was transferred to PHAA and only 14% of disappeared DOC was respired. The results imply that the aggregation process may favor the accumulation of amino acids more than respiration. The decrease in C- and N-yields in the treatment of AP indicated a preference for microbial consumption of DHAA. The C- and N-yields in the control and treatment of TR were higher than those in the initial, indicating that microorganisms preferentially use other DOM rather than DHAA.

Concentrations of PHNA in the control decreased from the initial, also indicating the microbial consumption of PHNA. A negative gross aggregation of PHNA occurred in AP. The gross aggregation of PHNA in AP was −43 nM, indicating that microorganisms consumed 18% more PHAA with the existence of aggregation compared to the control samples. Conversely, a positive gross aggregation of PHNA occurred in TR. The net aggregation of PHNA in TR was 128 nM, which accounted for an increase of 20.7% in the initial PHNA.

Concentrations of DHNA in the control of AP decreased from the initial, indicating that the microorganisms used DHNA as their carbon source. The DHNA in the treatment of AP was 95 nM lower than that in the control, indicating that the collision in the treatment group enhanced the microbial consumption of DHNA. The greater consumption of PHNA and DHNA in AP could be due to the limitation of organic carbon (particulate C/N ratio is <6 in the system). Neutral aldose served as a good carbon source for the microbial community. Contrary to AP, greater consumption of DHNA in the treatment has not been observed. The concentration of DHNA in the treatment of TR was similar to that in the control. The next question is which process contributes to the gross aggregation of 128 nM in PHNA? Bacterial colonization was ruled out due to the decrease in bacterial abundance. The phase transfer from the DHNA to the PHNA was more likely the possible process. However, the insignificant concentration difference in DHNA between the control and treatment groups remained unclear.

#### 2.2.3. Changes in Uncharacterized Organic Components

**Figure 1:**
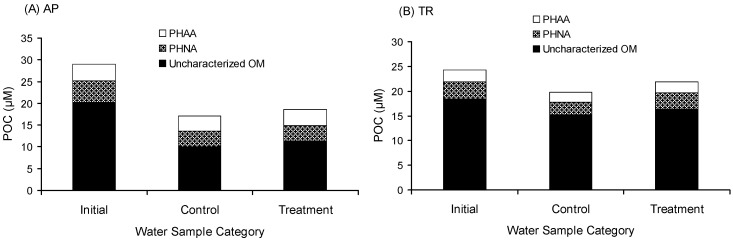
Chemical composition of particulate organic carbon (POC). (**A**) Avery Point station (AP); (**B**) Thames River station (TR). PHAA denotes particulate hydrolysable amino acids. PHNA denotes particulate hydrolysable neutral aldose. Uncharacterized OM denotes uncharacterized organic matter. Initial denotes the field data; treatment denotes samples after two days of rolling; and control denotes samples incubated for two days without rolling.

The collision mechanism also enhanced the accumulation of uncharacterized OM. The gross aggregation of uncharacterized OM in AP was 1.24 µM-C (Figure 1 and Table 1), which accounted for 4.3% of the initial POC. The gross aggregation of uncharacterized OM in TR was 1.12 µM-C (Figure 1 and Table 1), which accounted for 4.6% of the initial POC. The gross aggregation of uncharacterized OM is possibly the refractory DOM transferred to the particulate phase.

The uncharacterized DOM in the treatment was lower than that in the control. The uncharacterized DOM in the treatment of AP was 1.27 µM-C lower than that in the control, in which 97.6% was transferred to the particulate phase. The uncharacterized DOM in the treatment of TR was 36.25 µM-C lower than that in the control, in which only 3.1% was transferred to the particulate phase. About 97% of disappeared uncharacterized DOM (~35 µM-C) was respired, indicating that the aggregation process may enhance the uncharacterized DOM more biologically available. The gross aggregation of POC was predominantly uncharacterized. Uncharacterized POM accounted for 83% of the gross aggregation of POC in AP and 54% in TR, indicating that the aggregation process favors the accumulation of uncharacterized OM.

#### 2.2.4. Microbial Responses

In this study, bacterial abundance showed no significant difference among the initial, control, and treatment groups in both AP and TR stations, which was contradictory to previous findings of an increase of bacterial abundance during the aggregation of OM [32,33,34]. No significant change in bacterial abundance during the experiments could be due to the high mortality of bacteria from protozoa grazing and viral lysis [35,36]. The average protozoa grazing rate was 5.8 × 10^4^ cell bacteria m^−1^ h^−1^ in rivers and 2.9 to 4.7 × 10^4^ cell bacteria m^−1^ h^−1^ in seawaters [37]. The protozoa grazing could consume 5 to 250% of the bacterial population on a daily basis in marine waters [38]. Furthermore, viral lysis could also account for substantial bacterial mortality, up to 40% [39,40]. However, the planktonic community respirations (CR) did show differences (Table 4). Planktonic CR has been widely used to evaluate heterotrophic activity [41,42,43]. In general, bacterial respiration accounts for about 40% of CR; algal respiration contributes about 35% of CR; and the rest of 25% is consumed by metazoan heterotrophs [44]. In a close system, CR can be calculated by the change in total organic carbon (TOC = POC + DOC) divided by a unit of time (i.e., CR_control_ = (TOC_initial_ − TOC_control_)/Experimental duration; CR_treatment_ = (TOC_initial_ − TOC_treatment_)/Experimental duration). CR_control_ in coastal AP water (95 mg-C m^−3^ d^−1^) was lower than that in estuarine TR water (290 mg-C m^−3^ d^−1^; Table 4). CR_control_s were in a reasonable range. Previous studies reported that the CR could be more than 400 mg-C m^−3^ d^−1^ in the mouth of Yangtze River, China in early summer, where the salinity was ~30 psu [42], 49 to 3505 mg-C m^−3^ d^−1^ in Florida estuaries, USA [45], and 131 to 747 mg-C m^−3^ d^−1^ in Hudson River, NY, USA [46]. CR in treatment groups was higher than that in control groups (Table 4). CR_treatment_ was 5.4% higher than CR_control_ in AP and CR_treatment_ was 70.8% higher than CR_control_ in TR. The results indicated that the aggregation of OM enhanced subsequent microbial activity, which is consistent with previous findings [32,33], especially in estuarine TR waters.

The enrichment factor (EF) is a good aid in understanding a process effect for a specific component. EF could be calculated as $EF_{X,\;control}=\frac{{\left[X\right]}_{control}}{{\left[X\right]}_{initial}}$ and $EF_{X,\;treatment}=\frac{{\left[X\right]}_{treatment}}{{\left[X\right]}_{initial}}$.

If EF > 1, it indicates that the process tends to accumulate the given component. On the other hand, an EF < 1 indicates that the process favors the consumption of the given component. If EF = 1, it indicates that the process doesn’t favor either accumulation or consumption of the given component. EFs of PHNA are displayed in Table 5. In general, EF_control_s of PHNA were less than 1 in AP, except rhamnose and xylose, indicating microbial degradation of PHNA (Table 5). EFs in glucose and galactose in AP were lower than those in the other aldoses, indicating the selective microbial degradation of glucose and galactose. Because of the greater consumption of glucose and galactose, the mol fractions of glucose and galactose in control and treatment decreased from the initial in AP.

EF_control_s of PHNA were also less than 1 in TR (Table 5). The lowest EF in fucose indicated microbial selective degradation in fucose in TR. EF_treatment_ in galactose and glucose was larger than 1, indicating the accumulation of glucose and galactose. Because of the accumulation of glucose and galactose, the mol fractions of glucose and galactose in the treatment increased from the initial and control in TR. Microbes affected glucose and galactose in opposite directions in coastal AP waters and estuarine TR waters. This could be due to substrate dependency differing from system to system and leading to the great complexity of organic substrate dynamics.

## 3. Conclusions

The goal of his study was to investigate the collision effect on the aggregation of OM and the subsequent alterations of chemical characteristics in coastal and estuarine waters using a roller table. The mixed collision mechanism of shear and differential sedimentation enhanced the gross aggregation of POC, and the subsequent microbial degradation led to a negative net aggregation of POC in both AP and TR. Although bacterial abundance remained in a narrow range during the experiment, planktonic community respiration showed that aggregation of OM enhanced subsequent microbial activity. The collision is likely to aggregate biologically-labile organic matter (amino acids and neutral aldose in this study). However, in a C-limited system (low C/N ratio), neutral aldose is selectively consumed rather than aggregated. The collision also led to a gross aggregation of uncharacterized POM transferred from uncharacterized DOM. The aggregated, uncharacterized POM is possibly biologically refractory. EF analysis and C- and N-yields indicated that organic substrate dynamics are complicated.

## 4. Material and Methods

### 4.1. Study Sites and Field Sampling

**Figure 2:**
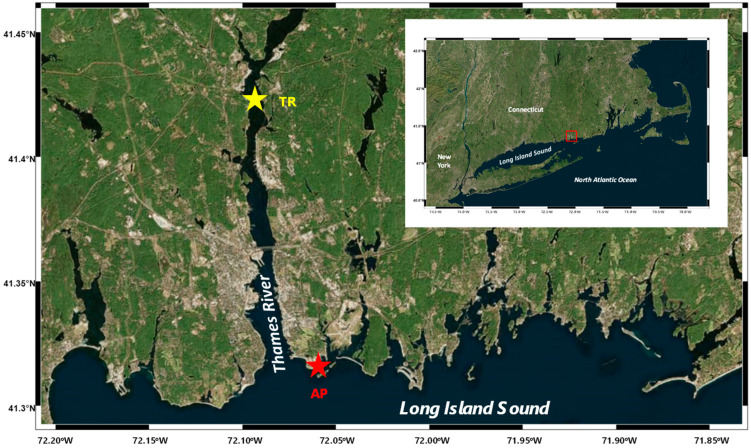
Study area and sampling sites. The red star denotes the coastal site of Avery Point (AP), while the yellow star denotes the estuarine site of the Thames River (TR).

Surface water samples were collected in May of 2010 at two sites in the eastern Long Island Sound, Connecticut, USA (Figure 2): (1) the coast of Avery Point, CT, USA (salinity ~30 psu; denoted AP); (2) the Thames River estuary in CT, USA (salinity ~5 psu; denoted TR). A handheld water meter (YSI 85, Yellow Springs, OH, USA) was used for the determination of in situ temperature and salinity. Water samples were collected using acid-washed 5-L Niskin bottles (General Oceanics, Miami, FL, USA), pre-filtered through a 200-µm-mesh net, stored in an acid-washed plastic bag, and transported in sheltered and cool conditions to the laboratory.

### 4.2. Experimental Design

The water samples were divided into three groups. In situ seawater was presented as the initial group. The experimental treatment consisted of seawater placed in 150-mL muffled glass bottles rotating on a roller table at a speed of 8 rpm (revolutions per minute) for 48 h in the dark. Seawater placed in 150 mL of muffled glass bottles for 48 h in the dark without rotation served as the control.

### 4.3. Chemical and Biological Determinations

A GF/F filter (Whatman, Chicago, IL, USA) with a nominal pore size of 0.7 µm was set as the operational cut-off of the particulate and dissolved forms. Particulate samples retained on individual pre-combusted GF/F filters were collected as replicate subsamples (100–150 mL) and kept frozen at −20 °C until the analysis. Dissolved samples were collected from the pre-combusted filtrate in 22-mL muffled glass vials and stored at −20 °C until the further analysis.

#### 4.3.1. Determination of POC and Particulate Organic Nitrogen (PON) Concentrations

After acidification with 10% HCl, POC, and PON concentrations were determined using a CHN elemental analyzer (Carlo-Erba, Waltham, MA, USA). POC and PON concentration determinations were standardized using a 7-point calibration curve of acetanilide (C_8_H_9_NO) in the range of 0.07 to 1.50 mg (R^2^ > 0.999). POC and PON concentrations were obtained by dividing the total C and N on each filter by the filtered volume of seawater. All POC and PON determinations were carried out in triplicate.

#### 4.3.2. Determination of DOC and Total Dissolved Nitrogen (TDN)

DOC concentrations were determined using the high temperature catalytic oxidation (HTCO) method on a TOC-V analyzer (Shimadzu, Kyoto, Japan) equipped with an automatic sample injection system [47]. DOC concentration determinations were standardized using a 7-point calibration curve of potassium hydrogen phthalate (C_6_H_4_·COOK·COOH) in the range of 0 to 500 µM-C (R^2^ > 0.999). All DOC concentration determinations were carried out in triplicate. TDN concentrations were determined simultaneously on a Shimadzu TOC-V analyzer equipped with a pyrochemoluminescence detector. TDN concentration determinations were standardized using a 7-point calibration curve of potassium nitrate solution in the range of 0 to 100 µM (R^2^ > 0.998). All TDN concentration determinations were carried out in triplicate.

#### 4.3.3. Determination of Amino Acids

Total hydrolysable amino acids (THAA) were hydrolyzed with concentrated HCl and determined on high-pressure liquid chromatography (HPLC; HP, Santa Clara, CA, USA) using a C-18 column and a methanol/acetonitrile mobile phase [48,49,50]. The procedure for hydrolysis and separation is described in detail in Svensson et al. (2004) [50]. In short, a 2 mL water sample (for dissolved hydrolysable amino acid determinations; DHAA) or a GF/F filter and 2 mL of Milli-Q water (for particulate hydrolysable amino acid determinations; PHAA), 2 mL of 10.5 M HCl, 20 µL of 11 mM ascorbic acid, and 12 µL of a mixed standard (DL-α-aminoadipic acid, DL-α-fluorophenylalanine, and 5-hydroxy-DL-lysine hydrate; 1 mM each) were added to a 10-mL ampoule. The ampoule was purged with nitrogen, sealed, and hydrolyzed at 110 °C for 24 h. Concentrations of amino acids were determined by HPLC with fluorescence detection after *O*-phthaldialdehyde derivatization. The amino acid concentration determinations were standardized using a 5-point calibration curve in the range 0.05 to 10 µM of each amino acid (R^2^ > 0.997). All amino acid determinations were carried out in triplicate.

#### 4.3.4. Determination of Neutral Aldoses

Concentrations of individual neutral aldoses were determined according to Skoog and Benner [51]. Briefly, 9 mL of filtered seawater (for dissolved hydrolysable neutral aldose determinations; DHNA) or a GF/F filter (for particulate hydrolysable neutral aldose determinations; PHNA) was evaporated to dryness, and 1 mL of 72 *w*/*w*% H_2_SO_4_ was added to the sample. Samples were placed in an ultrasonic bath for 15 min, and allowed to sit for an additional 1 h and 45 min. After this time, 9 mL of Milli-Q water was added to each sample, and the sample was then transferred to a muffled ampoule. The ampoule was placed in a dry bath at 100 °C for 3 h. The hydrolysis was terminated by placing the ampoule in an ice bath. Samples were neutralized by adding 1.44 g pre-combusted CaCO_3_ in small aliquots, and 200 µL of deoxyribose (final conc. 200 nM) was added as an internal standard. The sample was placed in an ultrasonic bath for 15 min, and the supernatant was separated by centrifugation. The collected supernatant was deionized using mixed anion (AG 2-X8, 20-50 mesh, Bio-Rad, Hercules, CA, USA) and cation (AG 50W-X8, 100–200 mesh, Bio-Rad) exchange resins. Concentrations of hydrolysable neutral aldose were determined by high-performance anion exchange chromatography with pulsed amperometric detection (HPAE-PAD) on a Dionex 500 system (Sunnyvale, CA, USA). The neutral aldose concentration determinations were standardized using a 5-point calibration curve in the range 0.1 to 1 µM for each aldose (R^2^ > 0.996). All neutral aldose determinations were carried out in triplicate.

#### 4.3.5. Determination of Inorganic Nutrients

Inorganic nutrients, including nitrate, nitrite, and phosphate, were determined for the dissolved fractions only. Nitrate concentrations and reduction with cadmium-copper filings to nitrite were determined using the diazo pink method [52]. Briefly, the produced nitrite reacts with sulfanilamide in an acid solution. The resulting diazonium compound was coupled with N-(1-naphthyl)-ethylenediamine dihydrochloride to form a colored azo dye, and the absorbance was measured at 550 nm. A 1000 mg L^−1^ stock solution of nitrate (Fisher Scientific, Waltham, MA, USA) was used to prepare an eight-point standard curve ranging from 0 to 40 µM-N on the day of analysis (R^2^ > 0.998). Artificial seawater (ASW) was used as a blank, preparing to a salinity of 30 psu using NaCl, MgSO_4_, NaHCO_3_, and Milli-Q water. Nitrite concentrations were determined in the same way as nitrate without the reduction process.

Phosphate concentrations were determined using the molybdenum-blue method [52]. Water samples were allowed to react with a composite reagent containing ammonium molybdate, ascorbic acid, and antimony potassium tartrate. The resulting complex (antimony-phospho-molybdate) was reduced to give a blue solution, and the absorbance was measured at 880 nm. A 1000 mg L^−1^ stock solution of phosphate (Fisher Scientific) was used to prepare a five-point standard curve ranging from 0 to 5 µM P on the day of analysis (R^2^ > 0.999). ASW, as described above, was used as a blank. All nutrient determinations were carried out on a SmartChem auto sampler (Westco^TM^, Milford, MA, USA).

#### 4.3.6. Determination of Bacterial Abundance

Bacterial abundance samples were collected in a 50-mL centrifuge tube, fixed with formaldehyde (final conc. 1%), stained with acridine orange, and collected on 0.2-µm pore size polycarbonate filters with pre-stained Irgalan-Black [53]. A direct count of bacterial abundance was then performed using epifluorescence microscopy (Zeiss, Oberkochen, Germany). All bacterial abundance samples were carried out in triplicate.

### 4.4. Data Analysis

JMP software (SAS, Cary, NC, USA) was used for statistical analysis. Data sets were tested for homogeneity of variance and then analyzed by means of a two-way analysis of variance (ANOVA) test using sample locations (AP and TR) and sample categories (Initial, Control, and Treatment) as independent variables. The models were examined for main effects as well as interaction effects. If the interaction term was significant, then the model was divided, and two one-way ANOVAs were used to examine differences between water sample categories within each location. If a one-way ANOVA was significant, then Tukey’s HSD test [54] was used to determine differences between water sample categories. Unless otherwise indicated, the variation around each mean is presented as +/− 1 standard deviation.

## Figures and Tables

**Table 1:** Aggregation of organic characteristics. Abbreviations: POC: particulate organic carbon; PON: particulate organic nitrogen; DOC: dissolved organic carbon; TDN: total dissolved nitrogen; PHAA: particulate hydrolysable amino acid; DHAA: dissolved hydrolysable amino acid; PHNA: particulate hydrolysable neutral aldose; AP: coastal Avery Point site; TR: estuarine Thames River site. Initial denotes the field data; treatment denotes samples after two days of rolling; and control denotes samples incubated for two days without rolling.

		AP			TR		
Parameter	Unit	Initial	Control	Treatment	Initial	Control	Treatment
POC	μM	28.97 ± 0.91	17.07 ± 0.63	18.57 ± 0.13	24.24 ± 0.80	19.86 ± 0.10	21.87 ± 1.07
PON	μM	5.10 ± 0.22	2.83 ± 0.28	3.19 ± 0.18	2.70 ± 0.14	2.33 ± 0.10	2.63 ± 0.11
Particulate C/N		5.56 ± 0.11	5.05 ± 0.43	5.85 ± 0.30	8.98 ± 0.41	8.51 ± 0.20	8.32 ± 0.24
DOC	μM	93.86 ± 4.96	89.94 ± 1.33	87.66 ± 4.27	209.11 ± 5.38	165.22 ± 15.91	128.88 ± 2.42
TDN	μM	7.07 ± 0.15	6.84 ± 0.76	6.15 ± 0.07	18.59 ± 0.27	14.54 ± 1.43	12.61 ± 0.28
Dissolved C/N		13.28 ± 0.42	13.28 ± 1.75	14.25 ± 0.77	11.25 ± 0.35	11.37 ± 0.24	10.36 ± 0.38
PHAA	nM	811 ± 22	725 ± 19	785 ± 25	510 ± 18	431 ± 17	474 ± 28
DHAA	nM	766 ± 41	586 ± 35	490 ± 27	626 ± 9	557 ± 9	507 ± 17
PHNA	nM	858 ± 37	615 ± 11	571 ± 22	617 ± 15	444 ± 12	572 ± 29
DHNA	nM	2370 ± 63	2247 ± 45	2152 ± 49	2962 ± 93	2880 ± 98	2910 ± 56

**Table 2:** Mol fraction of amino acids. Abbreviations: ALA: alanine; ARG: arginine; ASP: aspartic acid; GLU: glutamic acid; GLY: Glycine; HIS: histidine; ILE: isoleucine; LEU: leucine; MET: methionine; PHE: phenylalanine; SER: serine; THR: threonine; TYR: tyrosine; BALA: β-alanine; AP: coastal Avery Point site; TR: estuarine Thames River site. Initial denotes the field data; treatment denotes samples after two days of rolling; and control denotes samples incubated for two days without rolling. Each sample had triplicates.

Station	Sample	Group	ALA	ARG	ASP	GLU	GLY	HIS	ILE	LEU	MET	PHE	SER	THR	TYR	BALA
			(%)	(%)	(%)	(%)	(%)	(%)	(%)	(%)	(%)	(%)	(%)	(%)	(%)	(%)
AP	Particulate	Initial	10.6	6.7	13.3	11.7	11.0	1.8	4.6	7.9	6.7	4.6	7.5	8.1	0.6	4.9
		Control	9.8	6.9	13.3	11.4	11.0	2.1	4.7	8.3	6.8	4.6	7.6	7.7	0.8	5.0
		Treatment	10.5	6.4	13.7	11.7	11.2	2.0	4.8	8.4	6.8	4.6	7.7	7.7	0.3	4.2
	Dissolved	Initial	11.2	6.0	13.2	11.4	11.0	2.1	5.2	7.8	5.1	4.4	8.3	7.8	2.2	4.3
		Control	11.0	6.6	12.5	11.3	10.6	2.2	5.2	8.7	5.1	4.2	8.2	8.2	2.1	4.1
		Treatment	10.8	6.9	12.7	11.1	10.5	2.0	5.2	9.0	5.1	3.7	8.2	8.6	1.9	4.2
TR	Particulate	Initial	14.0	7.1	12.2	10.0	10.3	1.6	5.1	7.9	10.4	3.8	7.4	9.1	1.1	-
		Control	13.8	7.8	13.0	10.2	9.3	1.3	4.4	7.8	12.0	3.9	6.8	9.5	0.1	-
		Treatment	15.3	7.4	12.8	10.3	9.5	1.2	4.5	8.1	11.7	3.7	7.2	8.3	-	-
	Dissolved	Initial	11.6	6.1	15.3	9.6	11.7	3.6	6.0	8.0	4.5	3.9	8.2	6.6	1.9	2.9
		Control	11.7	6.4	15.0	9.5	11.4	3.3	5.5	8.2	5.0	4.0	8.2	7.3	1.7	2.8
		Treatment	11.9	6.0	14.8	9.4	12.0	3.0	5.0	8.2	6.0	4.0	8.3	7.0	2.0	2.5

**Table 3:** Mol fraction of neutral aldose. Abbreviations: AP: coastal Avery Point site; TR: estuarine Thames River site. Initial denotes the field data; treatment denotes samples after two days of rolling; and control denotes samples incubated for two days without rolling. Each sample had triplicates.

Station	Sample	Group	Fucose	Rhamnose	Arabinose	Galactose	Glucose	Mannose	Xylose
			(%)	(%)	(%)	(%)	(%)	(%)	(%)
AP	Particulate	Initial	9.4	7.0	8.4	20.8	35.8	9.5	9.0
		Control	10.6	9.9	10.7	17.7	23.3	11.5	16.3
		Treatment	10.5	9.5	10.4	17.3	24.5	11.3	16.7
	Dissolved	Initial	11.6	10.0	11.1	17.7	24.9	12.3	12.6
		Control	11.8	10.3	10.3	17.4	24.6	12.7	12.9
		Treatment	11.5	9.8	10.7	18.5	24.9	12.1	12.5
TR	Particulate	Initial	7.2	6.8	8.8	13.4	42.9	8.0	13.0
		Control	7.7	7.5	7.9	13.4	41.7	8.0	13.7
		Treatment	7.6	7.4	8.9	14.8	37.2	8.9	15.2
	Dissolved	Initial	8.1	9.3	7.0	12.5	37.4	15.5	10.3
		Control	8.0	9.1	6.9	12.7	38.4	15.0	9.8
		Treatment	8.7	9.7	7.3	12.7	35.6	15.8	10.2

**Table 4:** Planktonic community respiration of organic carbon. Abbreviations: AP: coastal Avery Point site; TR: estuarine Thames River site. Treatment denotes samples after two days of rolling, and control denotes samples incubated for two days without rolling.

Station	Planktonic Community Respiration (mgC m^−3^ d^−1^)	
	Control	Treatment
AP	94.9	100.0
TR	290.0	495.4

**Table 5:** Enrichment factor (EF) on particulate hydrolysable neutral aldose (PHNA). Abbreviations: AP: coastal Avery Point site; TR: estuarine Thames River site. Treatment denotes samples after two days of rolling, and control denotes samples incubated for two days without rolling.

	AP		TR	
	Control	Treatment	Control	Treatment
Fucose	0.80	0.74	0.54	0.68
Rhamnose	1.01	0.90	0.62	0.62
Arabinose	0.90	0.82	0.65	0.63
Galactose	0.60	0.55	0.76	1.23
Glucose	0.46	0.45	0.77	1.12
Mannose	0.87	0.78	0.75	0.84
Xylose	1.29	1.23	0.74	0.80
Overall	0.72	0.67	0.72	0.93

## Data Availability

The data presented in this study are available in the article.
